# The volume of steal phenomenon is associated with neurological deterioration in patients with large-vessel occlusion minor stroke not eligible for thrombectomy

**DOI:** 10.1177/23969873241251718

**Published:** 2024-05-14

**Authors:** Jacopo Bellomo, Martina Sebök, Christiaan HB van Niftrik, Vittorio Stumpo, Tilman Schubert, Jawid Madjidyar, Patrick Thurner, Christoph Globas, Susanne Wegener, Andreas R Luft, Zsolt Kulcsár, Luca Regli, Jorn Fierstra

**Affiliations:** 1 Department of Neurosurgery, University Hospital Zurich, Zurich, Switzerland; 2 Clinical Neuroscience Center (KNZ), Neuroscience Center (ZNZ), University of Zurich, Switzerland; 3 Department of Neuroradiology, University Hospital Zurich, Zurich, Switzerland; 4 Department of Neurology, University Hospital Zurich, Zurich, Switzerland; 5 Cereneo Center for Neurology and Rehabilitation, Vitznau, Switzerland

**Keywords:** Ischemic stroke, large-vessel occlusion, cerebrovascular reactivity, steal phenomenon, BOLD MRI, neurological deterioration

## Abstract

**Introduction:**

A significant number of patients who present with mild symptoms following large-vessel occlusion acute ischemic stroke (LVO-AIS) are currently considered ineligible for EVT. However, they frequently experience neurological deterioration during hospitalization. This study aimed to investigate the association between neurological deterioration and hemodynamic impairment by assessing steal phenomenon derived from blood oxygenation-level dependent cerebrovascular reactivity (BOLD-CVR) in this specific patient cohort.

**Patients and methods:**

From the database of our single-center BOLD-CVR observational cohort study (June 2015–October 2023) we retrospectively identified acute ischemic stroke patients with admission NIHSS < 6, a newly detected large vessel occlusion of the anterior circulation and ineligible for EVT. Neurological deterioration during hospitalization as well as outcome at hospital discharge were rated with NIHSS score. We analyzed the association between these two outcomes and BOLD-CVR-derived steal phenomenon volume through regression analysis. Additionally, we investigated the discriminatory accuracy of steal phenomenon volume for predicting neurological deterioration.

**Results:**

Forty patients were included in the final analysis. Neurological deterioration occurred in 35% of patients. In the regression analysis, a strong association between steal phenomenon volume and neurological deterioration (OR 4.80, 95% CI 1.32–31.04, *p* = 0.04) as well as poorer NIHSS score at hospital discharge (OR 3.73, 95% CI 1.52–10.78, *p* = 0.007) was found. The discriminatory accuracy of steal phenomenon for neurological deterioration prediction had an AUC of 0.791 (95% CI 0.653–0.930).

**Discussion:**

Based on our results we may distinguish two groups of patients with minor stroke currently ineligible for EVT, however, showing hemodynamic impairment and exhibiting neurological deterioration during hospitalization: (1) patients exhibiting steal phenomenon on BOLD-CVR imaging as well as hemodynamic impairment on resting perfusion imaging; (2) patients exhibiting steal phenomenon on BOLD-CVR imaging, however, no relevant hemodynamic impairment on resting perfusion imaging.

**Conclusion:**

The presence of BOLD-CVR derived steal phenomenon may aid to further study hemodynamic impairment in patients with minor LVO-AIS not eligible for EVT.

## Graphical abstract

**Graphical abstract:**
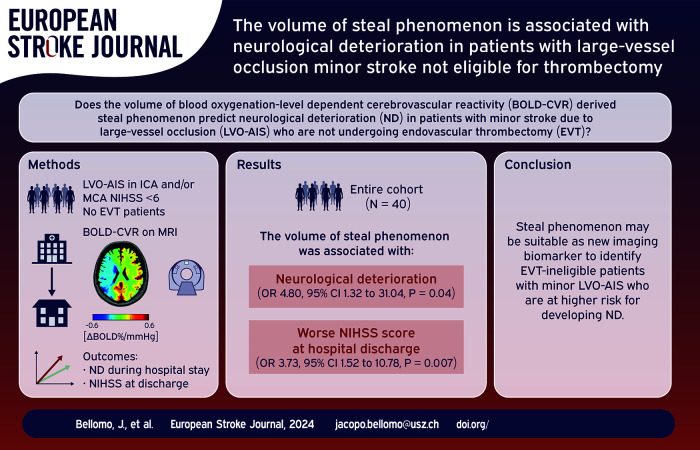

## Introduction

Large-vessel occlusion acute ischemic stroke (LVO-AIS) is mainly caused by cardio-aortic emboli and atherothrombotic disease, resulting in a high burden of disability.^1^ Timely recanalization of the blocked artery by means of endovascular thrombectomy (EVT) has shown an unequivocal improvement in clinical outcome, with patients` eligibility criteria for EVT ever expanding.^2,3^

The main goal of EVT is to mitigate irreversible ischemic brain tissue damage, which mainly depends on time since symptom onset and the compensatory capacity of the cerebral collateral circulation.^4^ Initially, the highest benefit of EVT was observed in patients presenting within 6 h from symptom onset with NIHSS score ⩾ 6 and ASPECTS score ⩾6.^2^ Recent trials,^2,3^ considering additional tissue perfusion imaging criteria for penumbra-core mismatch evaluation, have then extended this time window to 24 hours. Currently, EVT is recommended if patients have a NIHSS score ⩾ 6, infarct volume < 70 mL, penumbra-core mismatch volume ⩾ 15 mL, and penumbra-core mismatch ratio ⩾1.8.^5^

As a consequence, a significant number of patients presenting with mild symptoms (i.e. NIHSS < 6, “minor stroke”) are still excluded from receiving EVT but frequently experience early neurological deterioration.^6,7^ This urges further investigations into whether these patients exhibit hemodynamic impairment and may benefit from revascularization strategies,^8–10^ especially considering the unclear correlation of established resting perfusion imaging technique measurements with the neurological decline.^6^

Technological advancements in acute stroke hemodynamic imaging include blood oxygenation-level dependent cerebrovascular reactivity (BOLD-CVR), that has been validated as a novel imaging technique capable of assessing cerebrovascular vasodilatory reserve as the result of flow redistribution under a controlled hypercapnic challenge.^11,12^ Of particular relevance are brain regions exhibiting BOLD-CVR-derived steal phenomenon, reflecting a state of exhausted cerebrovascular reserve capacity, and indirectly indicating insufficient collateral compensation.^13,14^ Thus, BOLD-CVR could potentially be well suited to study brain tissue hemodynamic in this selected patient cohort.

In the present work, we studied the presence of BOLD-CVR steal phenomenon in patients with minor LVO-AIS not eligible for EVT to investigate its association with the occurrence of neurological deterioration during hospitalization, as well as the clinical status at hospital discharge.

## Materials and methods

### Study population

From our single-center BOLD-CVR observational cohort study database (June 2015–October 2023), we retrospectively identified acute ischemic stroke patients with admission NIHSS < 6 and symptoms correlating to a newly detected large vessel occlusion of the anterior circulation on CTA/MRA, who were considered ineligible for EVT and received both MR/CT perfusion and BOLD-CVR imaging in the acute phase of ischemic stroke (i.e. ⩽7 days from symptom onset time).^15^ The follow-up period corresponded to the duration of hospitalization following ischemic stroke. A detailed description of inclusion and exclusion criteria, EVT ineligibility criteria, baseline characteristics collection, and acute patient management are provided in the Supplemental Methods.

### Ethics

The Research Ethic Committee of the Canton Zurich, Switzerland (Kantonale Ethikkommission; KEK-ZH-NR. 2012-0427 and 2020-02314) approved the prospective BOLD-CVR data collection in a continuous database (June 2015–October 2023). Written informed consent was obtained from each participant before inclusion. The study was conducted in accordance with the ethical standards conform the latest standard set out by the Declaration of Helsinki.

### Outcome measures

The primary outcome measure was an acute neurological deterioration (ND) attributed to the underlying LVO during any point of hospitalization. ND was defined, according to Park et al.,^7^ as any new neurological worsening that satisfies 1 or more of the following criteria: an increase in total NIHSS score ⩾ 2, an increase in the NIHSS subscore 1a, 1b, or 1c (level of consciousness) ⩾1, or an increase in the NIHSS subscore 5a, 5b, 6a, or 6b (motor) ⩾1. Due to the exploratory nature of this study, a low NIHSS cut-off for neurological deterioration was chosen to capture all possible incidences. The secondary outcome measure was the NIHSS score at hospital discharge, or before undergoing delayed revascularization treatment if applicable.

### Image acquisition

Each patient received perfusion imaging, diffusion-weighted imaging (DWI), and BOLD-CVR imaging. Perfusion sequences were included in the standard stroke CT or MR imaging protocol at hospital admission. If initial evaluation involved CT, a follow-up MR examination, including diffusion-weighted imaging (DWI) to assess the infarct lesion, was conducted within 72 h from symptom onset time (or LVO detection time). BOLD-CVR imaging was performed either in the same or in a separate scanning session. Detailed information regarding BOLD-CVR imaging protocol and acquisition parameters are available in the Supplemental Methods.

### Image processing

Perfusion imaging data were analyzed with RAPID software (iSchemaView, Menlo Park, CA; https://www.rapidai.com/). The size of the penumbra was estimated from the volume of tissue for which there was delayed arrival of an injected tracer agent (time to maximum of the residue function, Tmax) exceeding 6 s. The size of core was automatically estimated from the perfusion data as CBF < 30%, in case of CTP, and from the diffusion-weighted imaging (DWI) as apparent diffusion coefficient (ADC) < 620 × 10^−6^ mm^2^/s, in case of MRP.

Infarct lesions were automatically segmented from DWI images (b0, b1000, and apparent diffusion coefficient map) using a deep learning-based algorithm published by Liu et al.,^16^ trained and tested in 2628 brain MRIs and publicly available at https://www.nitrc.org/projects/ads.

BOLD-CVR values, representing the BOLD signal percentage change during the hypercapnic stimulus, were calculated using a previously described analysis pipeline.^17^ Additionally, brain areas exhibiting steal phenomenon were isolated and quantified.^13^ This entailed selecting relevant voxels displaying a negative BOLD-CVR response (i.e. BOLD-CVR < 0% signal change/mmHg CO_2_), after subtracting the portion included in the infarct lesion. Unless specified otherwise, reported steal phenomenon volumes always refer to total volumes within ACA and MCA vascular territories.

### Statistical analysis

Statistical analysis was conducted using R studio (Posit Software, PBC formerly R Studio, version 02.07.2022). First, we looked at the association of primary and secondary outcome measures with steal phenomenon volume by means of logistic regression and ordinal logistic regression models. In both cases NIHSS values at hospital admission, DWI-derived infarct lesion size, and penumbra-core mismatch volume were included as covariates. Collinearity diagnostics were performed assessing correlation coefficients and variance inflation factors. To check if the proportional odds assumption was satisfied, we used the Brant-Wald test. Despite conducting BOLD-CVR imaging as close as possible to hospital admission, some patients underwent the examination after experiencing symptom deterioration. However, based on existing literature, we hypothesized that the presence of the steal phenomenon would predispose patients with LVO-AIS to a higher risk of neurological deterioration rather than being a consequence of it. Consequently, we conducted our statistical analysis without considering whether clinical deterioration occurred before or after the patients underwent the BOLD-CVR examination. To verify this assumption, we performed a subgroup analysis by excluding patients who had experienced clinical deterioration prior to BOLD-CVR imaging. Secondly, we performed receiver operating characteristic (ROC) analysis to assess the discriminatory accuracy of steal phenomenon volume for the primary outcome. All reported *p*-values are two-sided with significance level set at <0.05.

## Results

**Figure 1.**
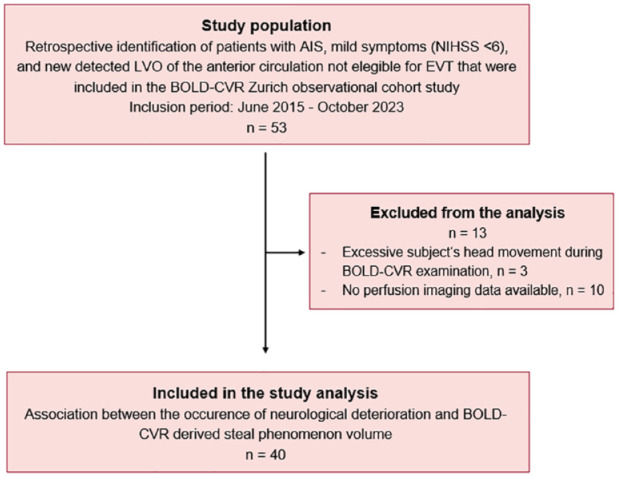
Study flow chart. BOLD-CVR: blood oxygenation-level dependent cerebrovascular reactivity; EVT: endovascular thrombectomy; LVO-AIS: large-vessel occlusion acute ischemic stroke.

Between June 2015 and October 2023, 53 patients with minor LVO-AIS who did not undergo endovascular thrombectomy received BOLD-CVR imaging as part of the baseline acute diagnostic examinations. Of these, 10 patients were excluded due to lacking perfusion imaging and 3 patients were excluded due to excessive head movement during BOLD-CVR imaging. The remaining 40 patients were included in the final analysis (Figure 1) and their baseline characteristics can be reviewed in Table 1.

**Table 1.** Demographic and clinical baseline characteristics.

Baseline characteristics	All (n = 40)
Age	
Median [IQR]	66.5 [59.8, 72.5]
Sex	
M (%)	27 (67)
F (%)	13 (33)
Ischemic event	
Stroke (%)	35 (87)
TIA (%)	5 (13)
Diseased vessels	
ICA (%)	31 (78)
MCA (M1 segment) (%)	7 (18)
Both (%)	2 (5)
Contralateral steno-occlusion (%)	8 (20)
NIHSS at first assessment	
Median [IQR]	1.0 [0.0, 2.0]
Comorbidities	
Atrial fibrillation (%)	2 (5)
Smoking history (%)	26 (65)
Hypertension (%)	31 (77)
Dyslipidemia (%)	20 (50)
Obesity (%)	4 (10)
Diabetes (%)	11 (27)
Coronary artery disease (CAD) (%)	3 (7)
Peripheral artery disease (PAD) (%)	4 (10)
Symptom onset time (SOT)	
Unclear SOT (%)	20 (50)
Symptom-to-imaging time^b^	
Symptom-to-perfusion (in h; mean (SD))	26.1 (25.3)
Symptom-to-BOLD (in h; mean (SD))	75.7 (44.4)
Reason for EVT ineligibility	
NIHSS < 6	40 (100)
Diffusion-weighted imaging	
Infarct lesion size (mL) (median [IQR])	2.1 [0.6, 11.1]
Perfusion imaging	
CTP (%)	10 (25)
MRP (%)	30 (75)
RAPID analysis^a^	
Core volume (mL) (median [IQR])	0.0 [0.0, 7.5]
Penumbra volume (mL) (median [IQR])	36.5 [0.0, 74.5]
Mismatch volume (mL) (median [IQR])	34.5 [0.0, 74.5]
Mismatch ratio > 1.8 (%)	22 (55)
BOLD-CVR imaging	
Steal phenomenon volume (mL) (median [IQR])	149.3 [56.8, 263.6]
IVT	
Yes (%)	2 (5)
No (%)	38 (95)
Antiplatelet treatment during follow-up	
Aspirin alone (%)	28 (70)
DAPT (%)	12 (30)

BOLD-CVR: blood oxygenation-level dependent cerebrovascular reactivity; CTP: computed tomography perfusion; DAPT: dual antiplatelet treatment; ICA: internal carotid artery; IVT: intravenous thrombolysis; MCA: middle cerebral artery; MRP: magnetic resonance perfusion; mRS: modified Ranking Scale; NIHSS: National Institutes of Health Stroke Scale; SOT: symptom onset time; TIA: transient ischemic attack.

Steal phenomenon volumes refer to total volumes found within ACA and MCA vascular territories.

a In four patients, RAPID analysis could not be performed due to low quality or incomplete perfusion imaging data.

b Symptom-to-imaging time is calculated only for patients with known symptom onset time.

The median [IQR] NIHSS score at first assessment was 1.0 [0.0, 2.0]. The mean (SD) time (in hours) from SOT to perfusion and BOLD-CVR imaging was 26.1 (25.3) and 75.7 (44.4), respectively. The median [IQR] infarct lesion size derived from DWI data was 2.1 [0.6, 11.1] mL. While the median [IQR] infarct core, penumbra and mismatch volume from the RAPID analysis were 0.0 [0.0, 7.5], 36.5 [0.0, 74.5], and 34.5 [0.0, 74.5] mL, respectively. The median [IQR] hospital stay was 8 [6, 10.5] days. No patient was lost to follow-up or died during hospitalization.

Overall, ND during hospitalization was observed in 14 patients (35%) of whom 6 had it before receiving BOLD-CVR examination. The severity of NIHSS worsening in these patients is illustrated in **Supplemental Figure 1.** Two patients showed only transient neurological worsening, while the other 12 experienced symptom persistence. Among them, 8 underwent follow-up MR examinations, confirming new lesions or infarct progression correlating with regions demonstrating BOLD-CVR steal phenomenon, while the remaining 4 did not undergo follow-up MRI. The median [IQR] NIHSS score at hospital discharge (or patient transfer for secondary intervention) was 1.0 [0.0, 3.0].

**Figure 2.**
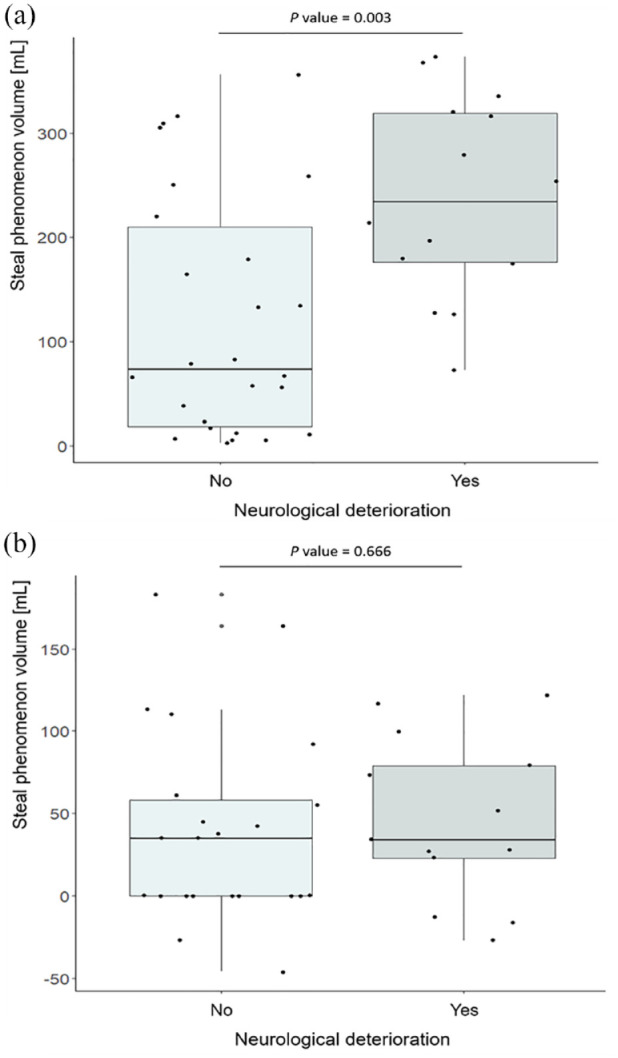
Comparison of the hemodynamic findings with respect to the occurrence of neurological deterioration. In panel A, the comparison of the BOLD-CVR-derived steal phenomenon volumes found in the MCA and ACA vascular territory are shown. In panel B, the comparison of the penumbra-core mismatch volumes are presented (in four patients, RAPID MR perfusion analysis could not be performed due to low quality perfusion imaging data). ACA: anterior cerebral artery; BOLD-CVR: blood oxygenation-level dependent cerebrovascular reactivity; MCA: middle cerebral artery. Between-group comparisons were made with Mann-Whitney *U*-test.

The BOLD-CVR and RAPID perfusion imaging findings among patients who did and did not develop ND are summarized in **Supplemental Table 1** and presented in Figure 2. Patients with ND showed a mean (SD) BOLD-CVR value in the MCA territory of −0.04 (0.07)% ΔBOLD/mmHg with a median [IQR] BOLD-CVR-derived steal phenomenon volume of 233.8 [176.2, 319.2] mL. While patients without symptom deterioration showed on average higher BOLD-CVR values (mean ± SD; 0.04 ± 0.09) and lower steal phenomenon volumes (median [IQR]; 72.8 [18.3, 210.0]). Regarding perfusion-derived mismatch volumes, patients who developed ND had a median [IQR] mismatch volume of 34.0 [23.0, 79.0] mL, while those who did not of 35.0 [0.0, 58.0] mL.

**Figure 3.**
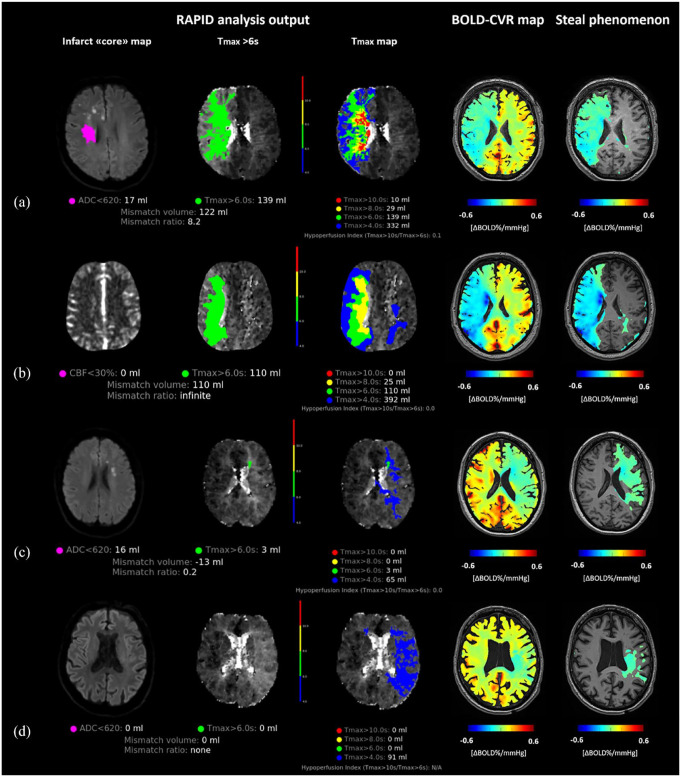
Illustrative patient cases. RAPID analysis and BOLD-CVR maps (including a whole-brain BOLD-CVR map and a BOLD-CVR map showing only steal phenomenon tissue areas) in four illustrative patients are presented. The first and second patients (panel A and B) both exhibit large steal phenomenon volume on BOLD-CVR, along with extended penumbra-core mismatch on MR perfusion imaging. While the first patient experienced neurological deterioration, the second did not. The third patient (panel C) demonstrates large steal phenomenon volume on BOLD-CVR, no penumbra-core mismatch, and experienced neurological deterioration. The fourth patient (panel D) had little steal phenomenon on BOLD-CVR, no mismatch volume, and did not show a deteriorating clinical course. ADC: apparent diffusion coefficient; BOLD-CVR: blood oxygenation-level dependent cerebrovascular reactivity; ICA: internal carotid artery; MCA: middle cerebral artery.

In the regression analyses (Table 2), larger steal phenomenon volume was significantly associated with both the occurrence of ND and higher NIHSS score at hospital discharge (or patient transfer for secondary intervention). This association held also in the subgroup analysis by excluding patients who had experienced clinical deterioration before undergoing BOLD-CVR imaging (**Supplemental Tables 2 and 3**). In Figure 3, four illustrative patient cases are presented.

**Table 2.** Regression analysis of the association between steal phenomenon volume and outcomes.

	Independent variable	Univariable OR (95% CI)	*p*-Value	Multivariable OR (95% CI)	*p*-Value
Neurological deterioration	Steal phenomenon volume (mL)	3.03 (1.44–7.44)	0.007*	4.80 (1.32–31.04)	0.04*
	Mismatch volume (mL)			0.99 (0.97–1.01)	0.54
	NIHSS at first assessment			1.30 (0.70–2.53)	0.40
	DWI-derived infarct lesion volume (mL)			1.05 (0.99–1.12)	0.03*
NIHSS score at discharge	Steal phenomenon volume (mL)	2.57 (1.36–5.13)	0.005*	3.73 (1.52–10.78)	0.007*
	Mismatch volume (mL)			0.99 (0.98–1.01)	0.44
	NIHSS at first assessment			2.30 (1.38–4.13)	0.002*
	DWI-derived infarct lesion volume (mL)			1.10 (1.04–1.18)	0.001*

Steal phenomenon volumes, encompassing total volumes within ACA and MCA vascular territories, were scaled prior to regression model fitting. The association between steal phenomenon and neurological deterioration was examined using logistic regression, while its association with NIHSS scores at discharge using ordinal logistic regression.*P* values that are statistically significant (<0.05) are bolded and marked with an asterisk (*).

BOLD-CVR: blood oxygenation-level dependent cerebrovascular reactivity; NIHSS: National Institutes of Health Stroke Scale.

**Figure 4.**
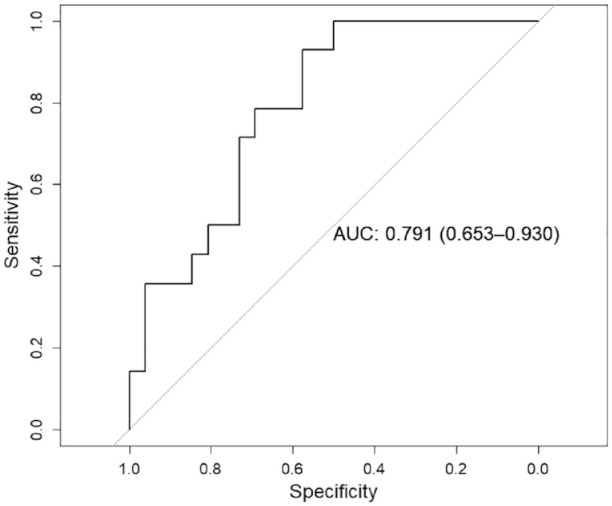
Discriminatory accuracy of steal phenomenon volume for neurological deterioration. Receiver operating characteristic (ROC) analysis showing the discriminatory accuracy of the extent of steal phenomenon volume for the prediction of neurological deterioration. Area under the curve (AUC) with 95% CI is reported.

In some patients who did not develop ND, we observed large steal phenomenon volumes comparable to those found in patients with symptom deterioration (Figure 3(a)). We investigated the discriminatory accuracy of steal phenomenon for neurological deterioration (Figure 4), and we found an AUC of 0.791 (95% CI 0.653–0.930). The largest Youden’s index was 0.51 for steal phenomenon volume >134 mL with sensitivity of 93% and specificity 58%.

## Discussion

In the present work, studying patients with mild symptoms (NIHSS < 6) due to large vessel occlusion acute ischemic stroke not eligible for endovascular thrombectomy, we found a significant association of larger BOLD-CVR-derived steal phenomenon volume with neurological deterioration during hospitalization as well as worse NIHSS score at hospital discharge. In contrast, the extent of penumbra-core perfusion mismatch volume did not show any association with the two clinical outcome measures.

Based on our results we may distinguish two groups of patients with minor stroke currently ineligible for EVT, however, showing hemodynamic impairment and exhibiting neurological deterioration during hospitalization:

(1) Patients exhibiting steal phenomenon on BOLD-CVR imaging as well as hemodynamic impairment on resting perfusion imaging.
(2) Patients exhibiting steal phenomenon on BOLD-CVR imaging, however, no relevant hemodynamic impairment on resting perfusion imaging.

These findings suggest that BOLD-CVR could potentially identify a larger patient group experiencing hemodynamic impairment and neurological deterioration compared to perfusion imaging alone. The observed discrepancy between perfusion findings and BOLD-CVR steal phenomenon in the second group may be explained by the compensatory effect of autoregulation mechanisms following hypoperfusion caused by LVO. This compensation may lead to a tissue hemodynamic state characterized by preserved or slightly reduced resting tissue perfusion but exhausted cerebrovascular reserve (i.e. oligemic hypoperfusion tissue state), which might remain undetected without the use of a vasodilatory stimulus.

### Current clinical acute ischemic stroke management of study population

Our study focused on minor stroke patients with LVO who did not satisfy the criteria of the current EVT guidelines. These patients presented by mild neurological symptoms and little infarct core volumes despite having large-vessel occlusion, suggesting that the occlusion likely resulted from a gradual acute-on-chronic process that allowed for the progressive development of collateral circulation.^18^ However, it has been reported that these patients often experience neurological deterioration during hospitalization, along with a higher rate of early stroke recurrence and poorer mid- and long-term clinical outcomes.^6,7^ These findings suggest that some patients may still suffer from significant hemodynamic impairment and could potentially benefit from thrombectomy or other reperfusion treatment strategies, such as EC-IC flow augmentation bypass surgery.^15,19^ In the past decade, hemodynamic imaging techniques, particularly perfusion imaging, have played a crucial role in the expansion of patients eligible for EVT.^20^ Of note, findings from the MR CLEAN LATE trial^21^ have underscored the value of angiographic studies alongside perfusion imaging in evaluating collateral status among LVO-AIS patients, thereby identifying additional candidates who may benefit from EVT. The diagnostic role of hemodynamic imaging techniques in patients with LVO-AIS relies on the fundamental concepts of stroke pathophysiology elucidated by positron emission tomography (PET). Following acute occlusion of a major intracranial artery, a hypoperfusion gradient emerges within the supplied vascular territory. Brain regions experiencing severe hypoperfusion rapidly progress to irreversible damage (“ischemic core”), while the remaining hypoperfused tissue, characterized by exhausted autoregulation, is divided into the “penumbra” and “oligemia” based on the severity of hypoperfusion.^22,23^ Tissue within the penumbra is functionally impaired and contributes to clinical deficits but remains potentially salvageable through effective reperfusion. Oligemic tissue experiences milder hypoperfusion with elevated cerebral blood volume and oxygen extraction fraction.^24^ Persistent occlusion, coupled with precipitating factors (such as systemic hypotension or anemia), may prompt oligemic tissue to transition into a penumbral state, leading to symptom deterioration.^22^

### Steal phenomenon and neurological deterioration

In our cohort, the observed incidence of ND (i.e. 35%) exceeded that reported in other studies.^6,7^ This may stem from our inclusion criteria, which focused solely on patients with LVO-AIS, and our definition of ND, which considered even moderate worsening of NIHSS scores. We found that the extent of steal phenomenon, rather than the volume of perfusion mismatch, correlated with both investigated outcome measures. To our knowledge, no other studies have explored BOLD-CVR findings in a similar study population. Previous studies have suggested a higher prevalence of fluctuating clinical courses and the appearance of new additional DWI lesions on follow-up imaging in LVO-AIS patients with large perfusion imaging mismatch volumes – a phenomenon known as the “total mismatch.”^25,26^ However, a recent work by Saleem et al.^6^ in a larger cohort found no significant association between the incidence of symptom deterioration and perfusion imaging measurements. Interestingly, we noted that some patients exhibiting large steal phenomenon volume did not experience ND. Supporting this observation, our analysis revealed that the ability of steal phenomenon to predict ND exhibited high sensitivity but scarce specificity. This underscores that in our cohort, patients lacking relevant steal phenomenon rarely encountered ND, whereas the presence of steal phenomenon did not always result in symptom deterioration. These findings support our hypothesis that steal phenomenon may indicate the correlate of an oligemic tissue state, wherein hypoperfusion induced by large-vessel occlusion can still be compensated through autoregulation under resting conditions. However, when secondary factors precipitate tissue perfusion, this hemodynamic state may become inadequate, leading to symptom worsening. Two potential mechanisms could precipitate tissue perfusion in the presence of steal phenomenon: (1)a hemodynamic-driven mechanism, where portions of the hypoperfused tissue with exhausted autoregulation fluctuate in and out of the penumbral state due to systemic changes in perfusion pressure^22^; (2) a thromboembolic-driven mechanism, where the compromised clearance ability of the distal bloodstream due to hypoperfusion hinders the removal of secondary emboli that originate from the occluded vessel.^27^

### Safety and feasibility

This work presents the first analysis of BOLD-CVR in a cohort of non-revascularized patients with LVO-AIS. Utilizing the infrastructure established at our institution, all included patients underwent BOLD-CVR and perfusion imaging examinations as part of their diagnostic work-up in the acute setting. No adverse events related to BOLD-CVR were observed. Only three out of the 53 patients who underwent BOLD-CVR experienced discomfort during the examination. However, in all cases, it was successfully resumed and completed during the same session. Three patients had to be discarded due to insufficient image quality caused by excessive head movement during the examination.

### Study limitations

First, the retrospective nature and the moderate sample size of this study may limit the generalizability of the results, necessitating validation in a prospective, larger cohort. Additionally, the clinical relevance of steal phenomenon on mid-term (90 days) and long-term (1 year) neurological outcomes requires further investigation. Furthermore, as mentioned above, some patients underwent the examination after experiencing symptom deterioration. Although a subgroup analysis excluding these patients showed similar results, ascertainment bias may have influenced our findings. Lastly, the use of two different modalities, MRP and CTP, to investigate perfusion tissue characteristics introduces an additional source of error, despite comparability between them has been shown.^28^

## Conclusion

BOLD cerebrovascular reactivity derived steal phenomenon is associated with neurological deterioration during hospitalization in patients with minor large-vessel occlusion acute ischemic stroke, who are not eligible for EVT. The presence of steal phenomenon may aid further investigation into hemodynamic impairment in this patient population.

## Supplemental Material

sj-docx-1-eso-10.1177_23969873241251718 – Supplemental material for The volume of steal phenomenon is associated with neurological deterioration in patients with large-vessel occlusion minor stroke not eligible for thrombectomy Supplemental material, sj-docx-1-eso-10.1177_23969873241251718 for The volume of steal phenomenon is associated with neurological deterioration in patients with large-vessel occlusion minor stroke not eligible for thrombectomy by Jacopo Bellomo, Martina Sebök, Christiaan HB van Niftrik, Vittorio Stumpo, Tilman Schubert, Jawid Madjidyar, Patrick Thurner, Christoph Globas, Susanne Wegener, Andreas R Luft, Zsolt Kulcsár, Luca Regli and Jorn Fierstra in European Stroke Journal
