# Supplementary material for: The volume of steal phenomenon is associated with neurological deterioration in patients with large-vessel occlusion minor stroke not eligible for thrombectomy
Source: Eur Stroke J. 2024 May 14;9(4):927–35. doi: 10.1177/23969873241251718 (PMC11569536; doi:10.1177/23969873241251718)
Supplement: sj-docx-1-eso-10.1177_23969873241251718 – Supplemental material for The volume of steal phenomenon is associated with neurological deterioration in patients with large-vessel occlusion minor stroke not eligible for thrombectomy [file sj-docx-1-eso-10.1177_23969873241251718.docx]

# SUPPLEMENTAL MATERIALS

**Supplemental Methods**

Inclusion and exclusion criteria

Inclusion criteria

1. Acute clinical ischemic stroke at hospital admission
2. First-ever LVO diagnosis of the anterior circulation, including occlusion of the internal carotid artery (ICA) and/or of M1-segment of the middle cerebral artery (MCA)
3. NIHSS score <6 at hospital admission and no eligibility for EVT (see below)
4. Infarct core volume <70 mL^1^
5. Maximal 7 days delay between completion of imaging examination and symptom stroke onset or, in case of unclear symptom onset time, LVO detection time
6. 18 years or above
7. Living independent before stroke (modified Ranking Scale, mRS ≤3)
8. Written informed consent of the patient or when the patient is not able to participate in the consenting procedure, the written authorization of an independent doctor who is not involved in the research project to safeguard the interests of the patients (in that case, post-hoc written informed consent of the patient or next of kin had to be obtained).

Exclusion criteria

1. Known chronic vessel occlusions
2. Nonthrombotic/embolic etiologies of the LVO such as Moyamoya syndrome or vasculitis
3. Major cardiac, psychiatric and/or neurological diseases
4. Early seizures
5. Known or suspected non-compliance, drug and/or alcohol abuse
6. Contra-indications for MRI
7. Documented evidence that the patient does not want to participate in any scientific study.

Ineligibility criteria for endovascular thrombectomy

In general, according to current guidelines^2^, patients were considered not eligible for EVT if they:

- Presented within 6 hours from ischemic symptom onset time with
  - NIHSS score <6 or ASPECTS <6
- Or presented between 6 and 24 hours from ischemic symptom onset time with
  - NIHSS score <6, infarct-core volume >70 mL, penumbra volume <15 mL or penumbra-core ratio <1.8

However, at our institution, patients with NIHSS <6 that present within 24h from symptom onset are treated in some selected situations. The decision to perform or not immediate EVT was evaluated case by case by both the senior neuroradiologist and neurologist on call.

Baseline characteristics

Demographic and clinical baseline characteristics were collected for each patient, encompassing stroke severity at hospitalization, symptom onset time, cerebrovascular risk factors (smoking history, dyslipidemia, obesity, diabetes mellitus, coronary artery disease, peripheral artery disease), history of atrial fibrillation, symptomatic side, and localization of the occluded vessel (ICA, M1-MCA, or both). Initial stroke severity, assessed by a certified physician upon arrival at our center, was determined using the National Institutes of Health Stroke Scale (NIHSS) and modified Rankin Scale (mRS) score. The diagnosis was categorized as either ischemic stroke or TIA, in accordance with the acute ischemic stroke definition set out by the American Heart Association.^3^

Acute stroke management

Usual care for patients with minor LVO-AIS who did not undergo EVT consisted of at least 24 hours of continuous monitoring and specialized care on a stroke unit. If after 24 hours patients were considered in a stable condition and not requiring intermediate care, they were transferred to post-stroke unit or regular ward care. In cases where underlying intracranial atherosclerotic disease (ICAD) was suspected, dual antiplatelet therapy (DAPT) was administrated according to our institutional guidelines. The diagnosis of ICAD was established based on classical risk factors and imaging criteria, such as eccentric stenosis/vessel wall enhancement, calcifications, and the presence of generalized atherosclerosis. When uncertainty arose, additional examinations were conducted to rule out other potential causes, such as vasculitis or dissection, through laboratory/CSF analyses, vessel wall MRI, and if necessary, DSA. In cases of symptom deterioration, blood pressure was maintained at permissive hypertensive values (i.e., systolic 160-180 mmHg) and the indication for delayed reperfusion treatment strategies was made based on interdisciplinary expert consensus.^4^ Furthermore, follow-up MR examination in these patients was considered by the treating physicians to assess the dynamics of tissue infarction associated with symptom deterioration.

BOLD-CVR imaging protocol

BOLD-CVR examination followed a previously published protocol^5^ and was conducted, either during the same or a separate scanning session to standard MR imaging, using a 3-Tesla Skyra MRI scanner (Siemens Healthineers, Forchheim, Germany) equipped with a 32-channel head matrix. Alongside the BOLD-CVR sequence, a high-resolution 3D T1-MPRAGE sequence was acquired as anatomical reference. A standardized carbon dioxide (CO_2_) stimulus was applied using the RespirAct™ (Thornhill Research Institute, Toronto, Canada). This involved 100 seconds at the patient specific resting P_ET_CO_2_, followed by an increase to 10 mmHg for 80 seconds, and then a return to resting P_ET_CO_2_ for 120 seconds, while P_ET_O_2_ levels were kept constant. Vital signs (including blood pressure, oxygen saturation, and electrocardiogram) were continuously monitored during the session to promptly address any potential adverse events. Moreover, NIHSS score was evaluated by a physician both before and after the BOLD-CVR scan to provide insights into the patients' neurological condition and any changes possibly induced by the imaging procedure.

Imaging acquisition parameters

TA: 6:46 min Coil Selection: Auto Voxel Size: 3.0×3.0×3.0 mm³ Acc:: 2 Rel. SNR: 1.00

**\\USER\test\test\CRPPstroke20191228_32ch_New_Patients6-dwicvrt1\CO2_bold ***

Properties

| Start measurement without further On preparation  Wait for User to Start Off  Start measurements Single Measurement  Prio Recon Off  Auto Open Inline Display Off  Auto Close Inline Display Off  Load Images to MR View&GO On  Auto Store Images On Load Images to Stamp Segments Off Load Images to Graphic Segments Off Graphic segment Default  Inline Movie Off |
| --- |

Routine

| Slice Group | 1 |
| --- | --- |
| Slices | 35 |
| Distance Factor | 10 % |
| Position | R0.8 P5.9 H10.6 mm |
| Orientation | T > C-16.5 > S0.6 |
| Phase Encoding Dir. | A >> P |
| Phase Oversampling | 0 % |
| FoV Read | 192 mm |
| FoV Phase | 100.0 % |
| Slice Thickness | 3.0 mm |
| TR | 2000.0 ms |
| TE | 30.00 ms |
| Averages | 1 |
| Concatenations | 1 |
| AutoAlign | Head > Brain |
| Coil Elements | HEA;HEP |

Contrast - Common

| TR 2000.0 ms  TE 30.00 ms  MTC Off  Flip Angle 85 deg  Fat-Water Contrast Fat Saturation  Reconstruction Magnitude |
| --- |

Contrast - Dynamic

| Dynamic Mode Standard  Measurements 200  Delay in TR 0.00 ms |
| --- |

Resolution - Common

| FoV Read 192 mm  FoV Phase 100.0 %  Slice Thickness 3.0 mm  Base Resolution 64  Phase Resolution 100 %  Interpolation Off |
| --- |

Resolution - Acceleration

| Acceleration mode GRAPPA  Reference Scans EPI/Separate  Acceleration Factor PE 2  Reference Lines PE 32  Phase Partial Fourier Off |
| --- |

Resolution - Filter

| Raw Filter Off  Elliptical Filter On  Hamming Off  Distortion Correction Off  Normalize Prescan |
| --- |

Geometry - Common

| Slice Group | 1 |
| --- | --- |
| Slices | 35 |
| Distance Factor | 10 % |
| Position | R0.8 P5.9 H10.6 mm |
| Orientation | T > C-16.5 > S0.6 |
| Phase Encoding Dir. | A >> P |
| Phase Oversampling | 0 % |
| FoV Read | 192 mm |
| FoV Phase | 100.0 % |
| Slice Thickness | 3.0 mm |
| TR | 2000.0 ms |
| Multi-Slice Mode | Interleaved |
| Series | Interleaved |
| Concatenations | 1 |

Geometry - AutoAlign

| Slice Group | 1 |
| --- | --- |
| Position Orientation  Phase Encoding Dir. | R0.8 P5.9 H10.6 mm T > C-16.5 > S0.6  A >> P |
| AutoAlign | Head > Brain |
| Initial Position | R0.8 P5.9 H10.6 |
| R | 0.8 mm |
| P | 5.9 mm |
| H | 10.6 mm |
| Initial Orientation | T > C |
| T > C | -16.50 |
| > S | 0.60 |
| Initial Rotation | -1.12 deg |

Geometry - Saturation

| Special Saturation None |
| --- |

Geometry - Tim Planning Suite

| Set-n-Go Protocol Off  Table Position 0 mm  Table Position H  Inline Composing Off |
| --- |

System - Miscellaneous

| Coil Selection Auto Coil Select  MSMA S - C - T  Sagittal R >> L  Coronal A >> P  Transversal F >> H  Coil Combination Adaptive Combine  Matrix Optimization Off |
| --- |

System - Adjustments

| Adjustment Strategy Standard  B0 Shim Standard  B1 Shim TrueForm  Adjustment Tolerance Auto |
| --- |

System - Adjustments

| Adjust with Body Coil Off  Confirm Frequency Never  Assume Silicone Off |
| --- |

System - Adjust Volume

| Position R0.8 P5.9 H10.6 mm  Orientation T > C-16.5 > S0.6  Rotation -1.12 deg  A >> P 192 mm  R >> L 192 mm  F >> H 116 mm  Reset Off |
| --- |

System - Tx/Rx

| Frequency 1H 123.256368 MHz  ? Ref. Amplitude 1H 0.000 V  Reset Off  Correction Factor 1.00  Image Scaling 1.000 |
| --- |

Physio - Signal

| 1st Signal/Mode None  TR 2000.0 ms  Concatenations 1 |
| --- |

BOLD

| GLM Statistics Off  Ignore Meas. at Start 0  Ignore After Transition 0  Model Transition States On  Temp. Highpass Filter On  Threshold 2.50  Paradigm Size 66  Meas[1] Active  Meas[2] Active  Meas[3] Active  Meas[4] Active  Meas[5] Active  Meas[6] Active  Meas[7] Active  Meas[8] Active  Meas[9] Active  Meas[10] Active  Meas[11] Active  Meas[12] Active  Meas[13] Active  Meas[14] Active  Meas[15] Active  Meas[16] Active  Meas[17] Active  Meas[18] Active  Meas[19] Active  Meas[20] Active  Meas[21] Active  Meas[22] Active  Meas[23] Ignore  Meas[24] Ignore  Meas[25] Ignore  Meas[26] Ignore  Meas[27] Ignore  Meas[28] Ignore  Meas[29] Ignore  Meas[30] Ignore  Meas[31] Ignore |
| --- |

BOLD

| Meas[32] Ignore  Meas[33] Ignore  Meas[34] Ignore  Meas[35] Ignore  Meas[36] Ignore  Meas[37] Ignore  Meas[38] Ignore  Meas[39] Ignore  Meas[40] Ignore  Meas[41] Ignore  Meas[42] Ignore  Meas[43] Ignore  Meas[44] Ignore  Meas[45] Active  Meas[46] Active  Meas[47] Active  Meas[48] Active  Meas[49] Active  Meas[50] Active  Meas[51] Active  Meas[52] Active  Meas[53] Active  Meas[54] Active  Meas[55] Active  Meas[56] Active  Meas[57] Active  Meas[58] Active  Meas[59] Active  Meas[60] Active  Meas[61] Active  Meas[62] Active  Meas[63] Active  Meas[64] Active  Meas[65] Active  Meas[66] Active  Motion Correction On  Spatial Filter On  Filter Width 4.0 mm  Measurements 200  Delay in TR 0.00 ms |
| --- |

Sequence - Part 1

| Sequence Name epfid  Excitation Standard  RF Pulse Type Normal  Gradient Mode Fast*  Bandwidth 2368 Hz/Px  Echo Spacing 0.53 ms  Free Echo Spacing Off  EPI Factor 64 |
| --- |

Sequence - Part 2

| Introduction Off |
| --- |

TA: 8:14 min Coil Selection: Auto Voxel Size: 0.8×0.8×1.0 mm³ Acc:: 2 Rel. SNR: 1.00

**\\USER\test\test\CRPPstroke20191228_32ch_New_Patients6-dwicvrt1\t1mprage_tra_HighRes_Nch_ 32ch ***

Properties

| Start measurement without further On preparation  Wait for User to Start Off  Start measurements Single Measurement  Prio Recon Off  Auto Open Inline Display Off  Auto Close Inline Display Off  Load Images to MR View&GO On  Auto Store Images On Load Images to Stamp Segments Off Load Images to Graphic Segments Off Graphic segment Default  Inline Movie Off |
| --- |

Routine

| Slab Group | 1 | |
| --- | --- | --- |
| Slabs | 1 |  |
| Distance Factor | 50 % |  |
| Position | L0.0 P1.6 H13.6 | mm |
| Orientation | Transversal |  |
| Phase Encoding Dir. | R >> L |  |
| Slices per Slab | 176 | |
| Phase Oversampling | 10 % | |
| Slice Oversampling | 27.3 % | |
| FoV Read | 230 mm | |
| FoV Phase | 100.0 % | |
| Slice Thickness | 1.0 mm | |
| TR | 2200.0 ms | |
| TE | 5.17 ms | |
| Averages | 1 | |
| Concatenations | 1 | |
| AutoAlign | Head > Brain | |
| Coil Elements | HEA;HEP | |

Contrast - Common

| TR 2200.0 ms  TE 5.17 ms  Magn. Preparation Non-sel. IR  TI 900 ms  Flip Angle 8 deg  Fat-Water Contrast Standard  Dark Blood Off  Reconstruction Magnitude |
| --- |

Contrast - Dynamic

| Dynamic Mode Standard  Measurements 1  Multiple Series Each Measurement  Reordering Linear Rot. |
| --- |

Resolution - Common

| FoV Read 230 mm  FoV Phase 100.0 %  Slice Thickness 1.0 mm  Base Resolution 288  Phase Resolution 100 %  Slice Resolution 100 %  Interpolation Off |
| --- |

Resolution - Acceleration

| Acceleration mode GRAPPA  Reference Scans Integrated  Acceleration Factor PE 2  Reference Lines PE 24  Acceleration Factor 3D 1  Phase Partial Fourier Off  Slice Partial Fourier Off  Asymmetric Echo Allowed  Elliptical Scanning Off |
| --- |

Resolution - Filter

| Raw Filter Off  Elliptical Filter Off  Distortion Correction 3D  Normalize Prescan  Image Filter On |
| --- |

Geometry - Common

| Slab Group | 1 |
| --- | --- |
| Slabs | 1 |
| Distance Factor | 50 % |
| Position | L0.0 P1.6 H13.6 mm |
| Orientation | Transversal |
| Phase Encoding Dir. | R >> L |
| Slices per Slab | 176 |
| Phase Oversampling | 10 % |
| Slice Oversampling | 27.3 % |
| FoV Read | 230 mm |
| FoV Phase | 100.0 % |
| Slice Thickness | 1.0 mm |
| TR | 2200.0 ms |
| Multi-Slice Mode | Single Shot |
| Series | Ascending |
| Concatenations | 1 |

Geometry - AutoAlign

| Slab Group | 1 |
| --- | --- |
| Position Orientation  Phase Encoding Dir. | L0.0 P1.6 H13.6 mm  Transversal R >> L |
| AutoAlign | Head > Brain |
| Initial Position | L0.0 P1.6 H13.6 |
| R | 0.0 mm |
| P | 1.6 mm |
| H | 13.6 mm |
| Initial Orientation | Transversal |
| Initial Rotation | 91.66 deg |

Geometry - Navigator Geometry - Tim Planning Suite

| Set-n-Go Protocol Off  Table Position 0 mm  Table Position H  Inline Composing Off |
| --- |

System - Miscellaneous

| Coil Selection Auto Coil Select  MSMA S - C - T |
| --- |

System - Miscellaneous

| Sagittal R >> L  Coronal A >> P  Transversal F >> H  Coil Combination Adaptive Combine  Matrix Optimization Off |
| --- |

System - Adjustments

| Adjustment Strategy Standard  B0 Shim Tune up  B1 Shim TrueForm  Adjustment Tolerance Auto  Adjust with Body Coil Off  Confirm Frequency Never  Assume Silicone Off |
| --- |

System - Adjust Volume

| Position Isocenter  Orientation Transversal  Rotation 0.00 deg  A >> P 263 mm  R >> L 350 mm  F >> H 350 mm  Reset Off |
| --- |

System - Tx/Rx

| Frequency 1H 123.256368 MHz  ? Ref. Amplitude 1H 0.000 V  Reset Off  Correction Factor 1.00  Image Scaling 1.000 |
| --- |

Physio - Signal

| 1st Signal/Mode None  TR 2200.0 ms  Concatenations 1 |
| --- |

Physio - Cardiac

| Fat-Water Contrast Standard  Magn. Preparation Non-sel. IR  TI 900 ms  Dark Blood Off  FoV Read 230 mm  FoV Phase 100.0 %  Phase Resolution 100 %  Dynamic Mode Standard |
| --- |

Physio - PACE

| Resp. Control Off  Concatenations 1 |
| --- |

Inline - Subtraction

| Subtract Off  Measurements 1  StdDev Off  Save Original Images On |
| --- |

Inline - Cardiac

Inline - MIP

| MIP Sag Off  MIP Cor Off  MIP Tra Off  MIP Time Off  Radial MIP Off  Save Original Images On  MPR Sag Off  MPR Cor Off  MPR Tra Off |
| --- |

Inline - Composing

| Inline Composing Off |
| --- |

Sequence - Part 1

| Sequence Name tfl_r  Dimension 3D  Excitation Slab-sel.  RF Pulse Type Fast  Gradient Mode Normal  Flow Compensation On  Reordering Linear Rot.  Bandwidth 250 Hz/Px  Echo Spacing 10.18 ms  Asymmetric Echo Allowed  Turbo Factor 317 |
| --- |

Sequence - Part 2

| Introduction On  RF Spoiling On  Incr. Gradient Spoiling On |
| --- |

Sequence - Assistant

| SAR Assistant Off |
| --- |

| Magn. Preparation Non-sel. IR  Save Original Images On  TE 5.17 ms  TR 2200.0 ms |
| --- |

**Supplemental Figures**

**Supplemental Figure 1. NIHSS score worsening in patients with neurological deterioration.**


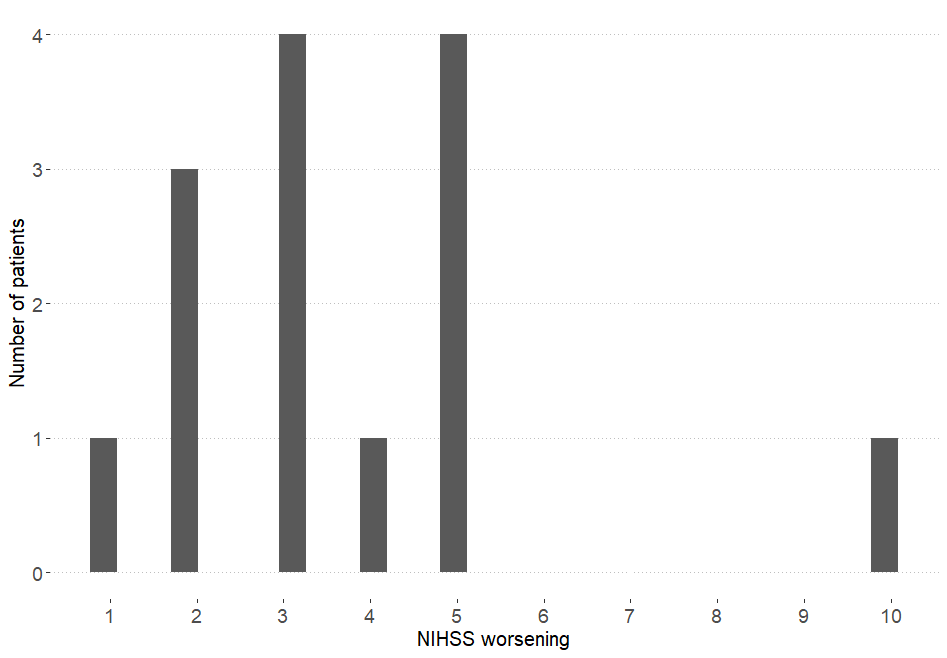


Caption: NIHSS, National Institutes of Health Stroke Scale.

**Supplemental Tables**

| **Imaging parameters** | **ND (n = 14/40)** | **Non-ND (n = 26/40)** | ***P* value** |
| --- | --- | --- | --- |
| **Diffusion-weighted imaging** |  |  |  |
| Infarct lesion size (mL) (median, [IQR]) | 9.30 [3.85, 17.10] | 0.90 [0.15, 3.42] | **0.001*** |
| **Perfusion imaging†** |  |  |  |
| Infarct core volume (mL) (median, [IQR]) | 6.00 [0.00, 17.00] | 0.00 [0.00, 0.00] | **0.014*** |
| Penumbra volume (mL) (median, [IQR]) | 58.00 [27.00, 79.00] | 35.00 [0.00, 58.00] | 0.080 |
| Mismatch volume (mL) (median, [IQR]) | 34.00 [23.00, 79.00] | 35.00 [0.00, 58.00] | 0.666 |
| Mismatch ratio >1.8 (%) | 11 (78%) | 15 (58%) | 0.234 |
| Tmax >4 sec. volume (mL) (median, [IQR]) | 184.00 [153.00, 276.00] | 140.00 [24.00, 255.00] | 0.134 |
| Tmax >6 sec. volume (mL) (median, [IQR]) | 58.00 [27.00, 79.00] | 35.00 [0.00, 58.00] | 0.080 |
| Tmax >10 sec. volume (mL) (median, [IQR]) | 0.0 [0.0, 0.0] | 0.0 [0.0, 0.0] | 0.447 |
| Hyperperfusion index (HI) (median, [IQR]) | 0.0 [0.0, 0.0] | 0.0 [0.0, 0.0] | 0.402 |
| **BOLD-CVR imaging** |  |  |  |
| BOLD-CVR values in MCA territory  (% ΔBOLD/mmHg) (mean (SD)) | -0.04 (0.07) | 0.04 (0.09) | **0.004*** |
| BOLD-CVR values in ACA territory  (% ΔBOLD/mmHg) (mean (SD)) | -0.01 (0.07) | 0.06 (0.07) | **0.007*** |
| Steal phenomenon volume in MCA territory  (mL) (median, [IQR]) | 186.77 [132.25, 230.79] | 63.50 [12.57, 160.55] | **0.004*** |
| Steal phenomenon volume in ACA territory  (mL) (median, [IQR]) | 58.09 [38.69, 89.01] | 12.95 [3.92, 52.02] | **0.004*** |
| Steal phenomenon volume in MCA and ACA territory  (mL) (median, [IQR]) | 233.81 [176.19, 319.19] | 72.82 [18.34, 209.95] | **0.003*** |

**Supplemental Table 1. BOLD-CVR and perfusion imaging characteristics.**

ACA, anterior cerebral artery; BOLD-CVR, blood oxygenation-level dependent cerebrovascular reactivity; IQR, interquartile range; MCA, middle cerebral artery; mL, millilitres; ND, neurological deterioration; SD, standard deviation. **†**In four patients RAPID analysis could not be performed due to low quality or incomplete perfusion imaging data. Between-group comparisons were made with Pearson χ2 or Fisher exact test, respectively Student t test or Mann-Whitney U test, as appropriate according to normality testing.

| **Independent Variable** | **Univariable OR**  **(95% CI)** | ***P* value** | **Multivariable OR**  **(95% CI)** | ***P* value** |
| --- | --- | --- | --- | --- |
| Steal phenomenon volume in MCA and ACA territory (mL) | 4.54 (1.67 to 18.31) | **0.01*** | 3.82 (0.77 to 41.83) | 0.09 |
| Mismatch volume (mL) |  |  | 1.00 (0.98 to 1.03) | 0.70 |
| NIHSS at first assessment |  |  | 1.47 (0.65 to 3.82) | 0.37 |
| DWI-derived infarct lesion volume (mL) |  |  | 1.17 (1.00 to 1.45) | 0.08 |

**Supplemental Table 2. Logistic regression analysis of the association between neurological deterioration and steal phenomenon volume in the subgroup of patients who developed neurological deterioration after receiving BOLD-CVR examination.**

Steal phenomenon volumes refer to total volumes found within ACA and MCA vascular territories and have been scaled before fitting logistic regression. ACA, anterior cerebral artery; BOLD-CVR, blood oxygenation-level dependent cerebrovascular reactivity; DWI, diffusion-weighted imaging; MCA, middle cerebral artery; mL, millilitres; NIHSS, National Institutes of Health Stroke Scale; OR, odds ratio.

| **Independent Variable** | **Univariable OR**  **(95% CI)** | ***P* value** | **Multivariable OR**  **(95% CI)** | ***P* value** |
| --- | --- | --- | --- | --- |
| Steal phenomenon volume in MCA and ACA territory (mL) | 2.13 (1.12 to 4.26) | **0.009*** | 3.10 (0.93 to 14.77) | 0.09 |
| Mismatch volume (mL) |  |  | 1.00 (0.97 to 1.02) | 0.94 |
| NIHSS at first assessment |  |  | 2.89 (1.46 to 7.14) | **0.007*** |
| DWI-derived infarct lesion volume (mL) |  |  | 1.23 (1.09 to 1.42) | **0.001*** |

**Supplemental Table 3. Ordinal logistic regression analysis of the association between NIHSS score at hospital discharge and steal phenomenon volume in the subgroup of patients who developed neurological deterioration after receiving BOLD-CVR examination.**

Steal phenomenon volumes refer to total volumes found within ACA and MCA vascular territories and have been scaled before fitting logistic regression. ACA, anterior cerebral artery; BOLD-CVR, blood oxygenation-level dependent cerebrovascular reactivity; DWI, diffusion-weighted imaging; MCA, middle cerebral artery; mL, millilitres; NIHSS, National Institutes of Health Stroke Scale; OR, odds ratio.

**References**

1. Albers GW, Marks MP, Kemp S, et al. Thrombectomy for Stroke at 6 to 16 Hours with Selection by Perfusion Imaging. *N Engl J Med*. 2018;378(8):708-718. doi:10.1056/NEJMoa1713973

2. Powers WJ, Rabinstein AA, Ackerson T, et al. Guidelines for the Early Management of Patients With Acute Ischemic Stroke: 2019 Update to the 2018 Guidelines for the Early Management of Acute Ischemic Stroke: A Guideline for Healthcare Professionals From the American Heart Association/American Stroke Association. *Stroke*. 2019;50(12):e344-e418. doi:10.1161/STR.0000000000000211

3. Sacco RL, Kasner SE, Broderick JP, et al. An updated definition of stroke for the 21st century: a statement for healthcare professionals from the American Heart Association/American Stroke Association. *Stroke*. 2013;44(7):2064-2089. doi:10.1161/STR.0b013e318296aeca

4. Sebök M, Esposito G, Niftrik CHB van, et al. Flow augmentation STA-MCA bypass evaluation for patients with acute stroke and unilateral large vessel occlusion: a proposal for an urgent bypass flowchart. *J Neurosurg*. Published online January 7, 2022:1-9. doi:10.3171/2021.10.JNS21986

5. van Niftrik CHB, Piccirelli M, Bozinov O, et al. Iterative analysis of cerebrovascular reactivity dynamic response by temporal decomposition. *Brain Behav*. 2017;7(9):e00705. doi:10.1002/brb3.705
